# Transcription of a B chromosome *CAP-G* pseudogene does not influence normal Condensin Complex genes in a grasshopper

**DOI:** 10.1038/s41598-017-15894-5

**Published:** 2017-12-15

**Authors:** Beatriz Navarro-Domínguez, Francisco J. Ruiz-Ruano, Juan Pedro M. Camacho, Josefa Cabrero, María Dolores López-León

**Affiliations:** 1 Departamento de Genética. Facultad de Ciencias, Universidad de Granada, 18071 Granada, Spain; 2 Department of Ecology, Evolution, and Organismal Biology, Iowa State University, Ames, Iowa USA

## Abstract

Parasitic B chromosomes invade and persist in natural populations through several mechanisms for transmission advantage (drive). They may contain gene-derived sequences which, in some cases, are actively transcribed. A further interesting question is whether B-derived transcripts become functional products. In the grasshopper *Eyprepocnemis plorans*, one of the gene-derived sequences located on the B chromosome shows homology with the gene coding for the CAP-G subunit of condensin I. We show here, by means of fluorescent *in situ* hybridization coupled with tyramide signal amplification (FISH-TSA), that this gene is located in the distal region of the B24 chromosome variant. The DNA sequence located in the B chromosome is a pseudogenic version of the *CAP-G* gene (*B-CAP-G*). In two Spanish populations, we found active transcription of *B-CAP-G*, but it did not influence the expression of *CAP-D2* and *CAP-D3* genes coding for corresponding condensin I and II subunits, respectively. Our results indicate that the transcriptional regulation of the *B-CAP-G* pseudogene is uncoupled from the standard regulation of the genes that constitute the condensin complex, and suggest that some of the B chromosome known effects may be related with its gene content and transcriptional activity, thus opening new exciting avenues for research.

## Introduction

Chromatin condensation and chromosome segregation during cell division are two essential events for maintenance and transmission of genetic information. Condensin complexes play an important role in these processes^1,2^, which explains the growing interest in their study. Condensins are highly conserved heteropentameric complexes, constituted by a V-shaped dimer of SMC (structural maintenance of chromosomes) ATPase core subunits and an additional set of non-SMC regulatory CAP (chromosomal associated protein) subunits. Most eukaryotes contain two different types of condensin complexes, known as condensins I and II, which accomplish different functions and are subjected to different regulation during mitosis and meiosis. Condensins I and II share the same heterodimeric pair of SMC subunits (SMC2 and SMC4), and are composed of different kleisin subunits (CAP-H and CAP-H2, respectively) and HEAT-repeat subunits (CAP-D2/CAP-G for condensin I and CAP-D3/CAP-G2 for condensin II)^3^.

The most conspicuous phenotype of condensin mutants is the massive formation of bridges between chromosomes during mitotic and meiotic anaphases, as a consequence of defective resolution of concatenations between sister chromatids and incorrect compaction of chromosomes prior to mitotic anaphase separation^1^. Anaphase bridges are associated with DNA double strand breaks^4^, which often result in chromosome rearrangements such as translocations or deletions^5^ and also gene amplification through the breakage-fusion-bridge (BFB) cycle^4,6^. The absence of condensin also entails fuzzy chromosome appearance (for review, see Hirano^7^). Furthermore, condensins have a role in other cellular processes, as suggested by the fact that in *C. elegans*, mutants for any condensin I subunit show changes in DNA double-stranded break distribution and frequency, causing a boost in the frequency of crossover events^8^.

Effects of mutations on the *CAP-G* subunit gene in *Drosophila* have been profusely described in the literature, thus providing many insights on its role in the overall function of the condensin complex. Some of these effects are embryonic lethality, female infertility, delays in chromosome condensation during prophase, failures in sister chromatid separation and resolution, lagging chromosomes, bridging, and ultimately aneuploidy. *CAP-G* also has a role in gene expression regulation during interphase, possibly associated with the suppression of position-effect variegation^9^. Furthermore, the CAP-G subunit has been shown to interact not only genetically but also physically with CID, the *Drosophila* homolog for CENP-A, evidencing a link between condensin and kinetochore structure^10^.

Many eukaryote genomes harbor special additional and dispensable chromosomes called supernumerary (B) chromosomes (for review, see^11–13^). They are considered intragenomic parasites taking advantage of cell replication machinery for its accumulation and persistence in natural populations. They do not obey Mendelian segregation, managing to incorporate into gametes at rates higher than 0.5. This alteration of the normal process of cell division (known as drive) allows a rapid increase in B chromosome frequency in natural populations. Drive mechanisms can operate before, during or after meiosis^14^, but how the alteration of cell division is controlled by the B chromosome is mostly unknown^15^. In rye, the nondisjunction of the B chromosome during first mitotic division during pollen maturation is likely caused by extended cohesion of the B sister chromatids^16^. The presence of genes with functions related to cell division in B chromosomes (like the B-CAP-G pseudogene reported here) opens the possibility to elucidate the molecular aspects of this interesting interaction between standard and parasitic chromosomes.

Most B chromosomes are heterochromatic and mainly composed of non-coding repetitive DNA. Nevertheless, protein-coding genes have recently been found in B chromosomes from several animal and plant systems^17–24^. In addition, transcription of B-chromosome sequences has been reported in several organisms such as rye^23,25^, maize^22^ and Siberian roe deer^26^. In the grasshopper *Eyprepocnemis plorans*, the B chromosome variant named B24 harbors ribosomal RNA genes expressed at low rates^27–29^, at least four protein-coding genes containing their complete coding sequence (CDS), and six other genes with fragmented CDSs^24^. One of these fragmented genes shows homology with the *CAP-G* subunit of condensin I. In contrast to the *CAP-G* sequence found in the *Locusta migratoria* draft genome^30^, the *CAP-G* sequence found in the B24 chromosome (from here onwards, *B-CAP-G*) of *E. plorans* lacks the last five exons (20–24) of the 3′ end. Remarkably, the *B-CAP-G* gene is actively transcribed in B24-carrying adult males and females from the Torrox population^24^.

It is not known how the B chromosomes of *E. plorans* control their own drive, but they could take advantage of alterations in the correct functioning of cell division, which would allow to bypass the mitotic and/or meiotic checkpoints. The expression of the B-CAP-G pseudogene could be very interesting at this respect. Several papers have suggested the possible implication of condensins in the spindle assembly checkpoint (SAC)^31–33^. Furthermore, a small fraction of condensin I remains in the cell nucleus during interphase where it acts on gene regulation. Mutants fail in activating cellular control pathways^34,35^.

Due to the high relevance of the condensin complex, and its possible impact on B chromosome maintenance in natural populations, we analyze here the changes in DNA sequence shown by the *B-CAP-G* pseudogene found in B chromosomes from two populations (Salobreña and Torrox) of the grasshopper *E. plorans*, where the predominant B chromosome variants are B2 and B24, respectively^36^. These two B variants are mainly composed of a 180 bp satellite DNA and ribosomal DNA. They show differences in size and relative amount of these two sequences, with B24 being derived from B2^37,38^. Likewise, we analyze the transcriptional activity of the *B-CAP-G* pseudogene and its possible influence on the activity of other condensin complex protein subunit genes (*CAP-D2* and *CAP-D3*), as an indirect indication for its possible functional role.

## Results

### Tyramide-coupled FISH reveals B-CAP-G pseudogene localization in the distal region of the B24 chromosome

**Figure 1:**
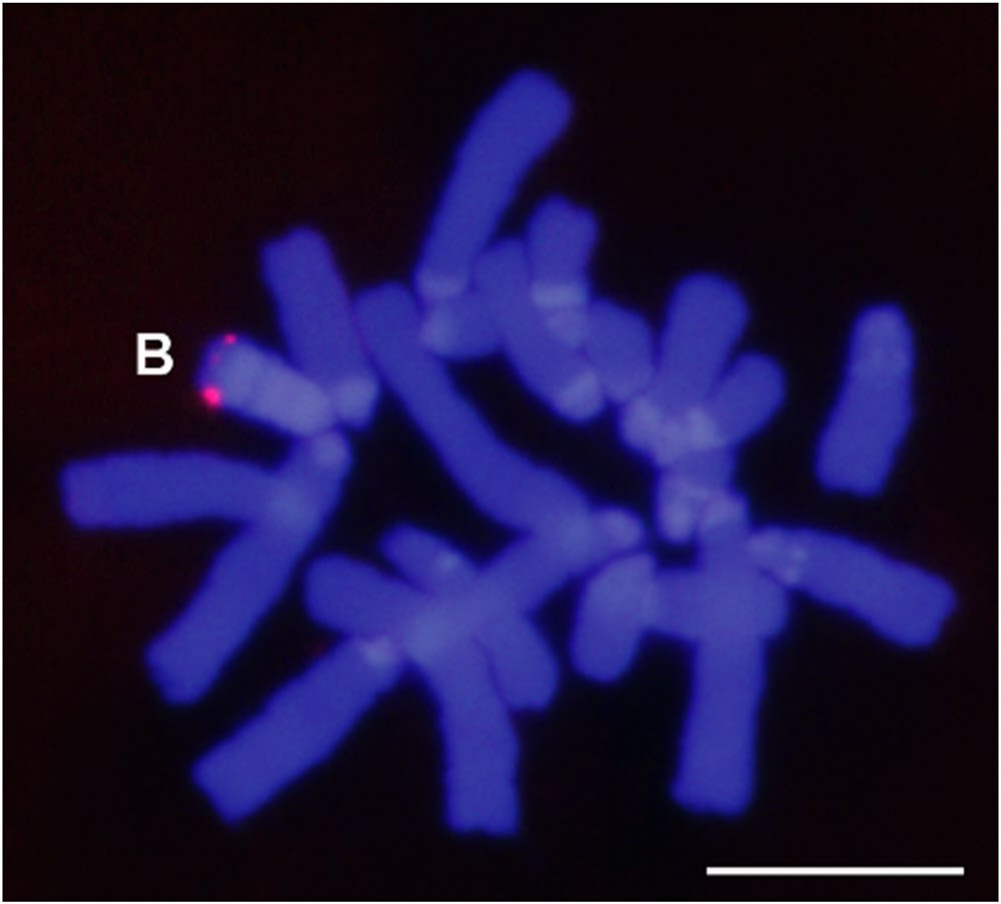
FISH-TSA showing the physical location of the *B-CAP-G* pseudogene in the distal region of the B24 chromosome. The absence of signal on A chromosomes suggests a tandem organization of several copies of this pseudogene in the B chromosome. Bar = 10 µm.

The FISH-TSA experiments showed that the B24 chromosome in the Torrox population carries *B-CAP-G* pseudogenes in the distal region (Fig. 1). No consistent signal was found on A chromosomes. This difference is explained by the presence of about twelve copies of the pseudogene per B24 chromosome^24^.

### The B-CAP-G pseudogene of the B24 chromosome shows specific sequence changes and is transcribed

**Figure 2:**
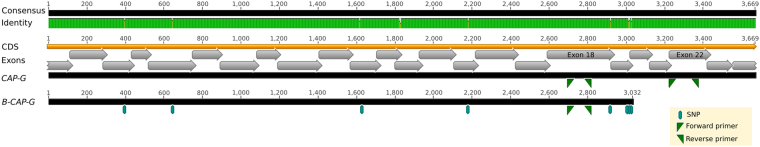
Diagram comparing CDSs of *E. plorans CAP-G* and *B-CAP-G* sequences. Note that five last exons (20 to 24) are missing in the pseudogenic *B-CAP-G* sequence. Primers for qPCR experiments were designed previously in Navarro-Domínguez *et al*.^24^, anchoring in exon 18 (present in both *CAP-G* and *B-CAP-G* sequences) and exon 22 (only in *CAP-G* sequence). Nucleotidic changes associated with B chromosome presence are marked in the diagram and further described in Fig. 3.

The CDS of the complete *CAP-G* transcript (discarding the UTR regions) found in B-lacking *E. plorans* individuals was 3,669 bp long, thus showing the same length as that found in *L. migratoria*, but being 15 nucleotides shorter than that found in *Chorthippus mollis* (3,684 bp). *E. plorans* standard *CAP-G* gene is presumably structured into 24 exons, likewise the gene present in the *L. migratoria* genome^30^. The *B-CAP-G* pseudogene found in B24 variant (Torrox), however, lacks the last five 3′ exons (exons 20–24; Fig. 2). This is shown by the coverage pattern of the genomic reads from a 4B male^24^. Therefore, the *B-CAP-G* pseudogene is about 18% shorter than the *CAP-G* gene found in A chromosomes.

**Figure 3:**
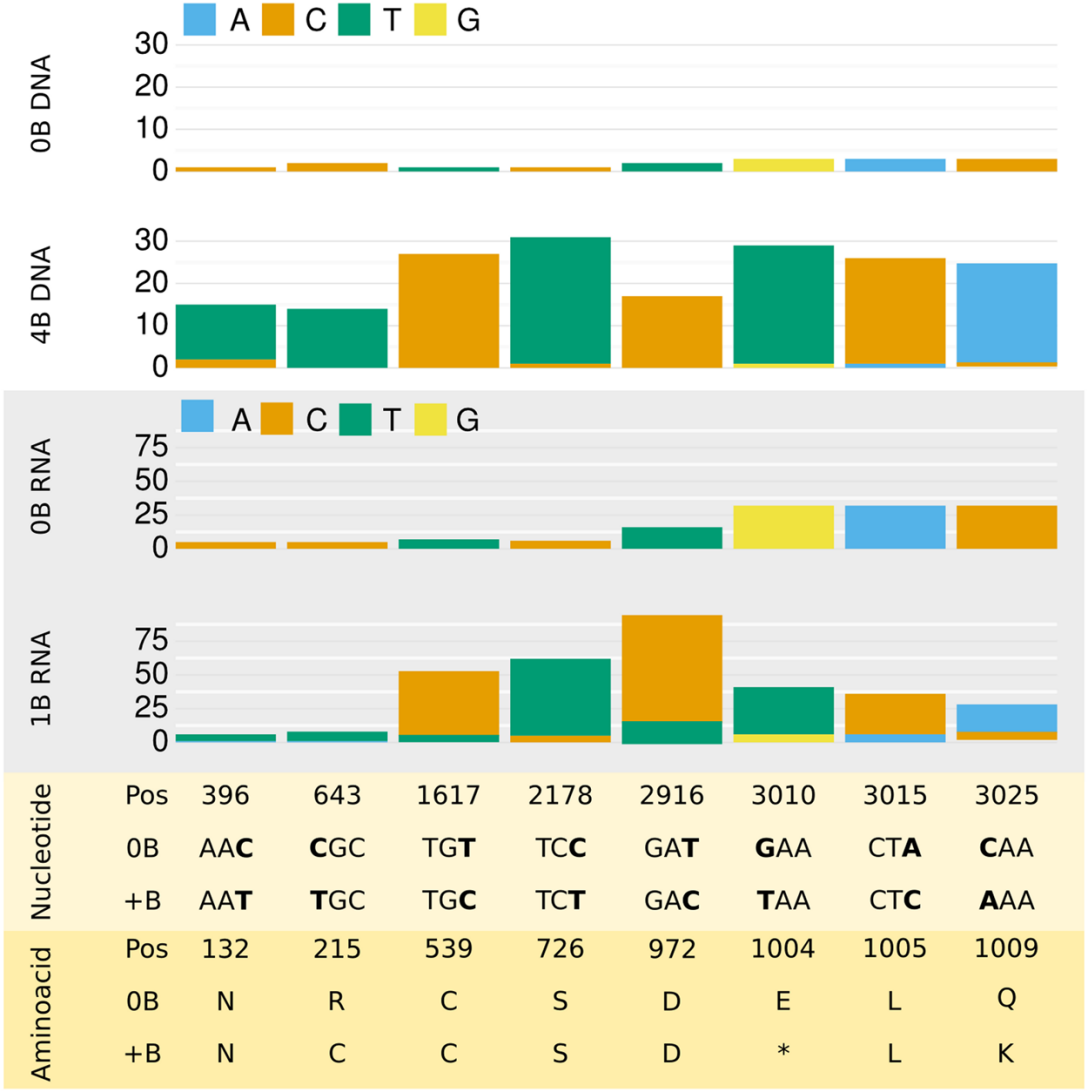
Nucleotidic variation in the *CAP-G* gene associated with B chromosome presence in *E. plorans* from Torrox population. The upper panel (white background) shows gDNA counts for 8 nucleotidic positions in 0B and 4B males from Torrox. Note that the 0B reference is usually at lower frequency because of the presence of several gene copies in the B chromosome. The middle panel (grey background) shows RNA counts for these same 8 nucleotidic positions in 0B and 1B females from Torrox. Note that the 0B female has the same nucleotides as the 0B gDNA from Torrox, and that the 1B female has esentially the same nucleotide composition as the B-carrying gDNA libraries, indicating the expression of B chromosome gene copies. The lower panel shows codon (light yellow background) and aminoacidic (yellow background) changes provoked by the 8 substitutions. Note that only nucleotidic changes in positions 643 and 3,010 provoke alterations on the predicted CDS of the B chromosome gene copies (R for C in the 215 residue and E for stop codon in the 1004 residue, respectively), and that the substitution of nucleotide 3,025 is beyond the stop codon.

Remarkably, B-carrying individuals from this same population showed that *B-CAP-G* transcript amount increased with B chromosome number when probed for exon 18, but not for exon 22, thus indicating active transcription of the *B-CAP-G* pseudogene^24^. Sequence analysis on gDNA and RNA showed the presence of eight nucleotide changes being exclusive of B-carrying libraries from Torrox, both in gDNA and RNA, further demonstrating the expression of the B-CAP-G pseudogene (Figs 2 and 3). Five of these substitutions were synonymous and thus lack apparent impact on the predicted protein. However, one of the three remaining substitutions was a non-synonymous transition of C to T in position 643 of the CDS (exon 5), changing a CGC codon (Arg) to TGC (Cys). Another substitution was a transversion from G to T at position 3,010 (exon 19 i.e. the last codon before gene truncation on the B chromosome), changing GAA (Glu codon) to TAA (stop codon), thus shortening the predicted protein in 199 amino acids from the C-terminus. Finally, we found another non-synonymous substitution at position 3,025 (CAA to AAA, changing Gln to Lys), but it is located beyond the premature stop codon and thus probably lacks impact on the predicted protein. Therefore, the predicted protein for the *B-CAP-G* transcript B24 is 1,004 amino acids long, instead of the 1,223 of that expected from the A chromosome transcript.

**Figure 4:**
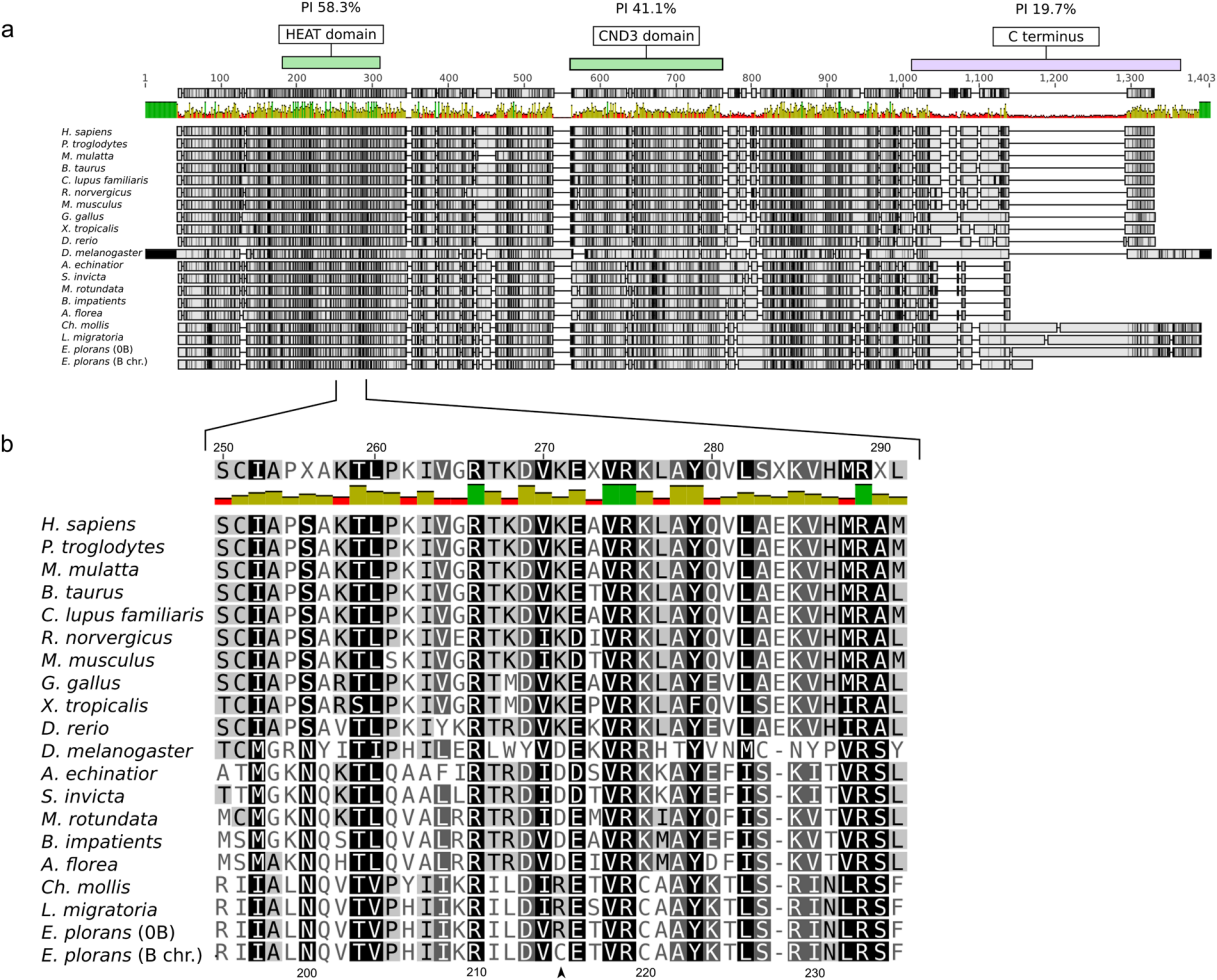
Protein alignment of the complete sequence (**a**) and the conserved HEAT domain (**b**) of CAP-G from several organisms. (**a**) Note that the predicted protein generated if the B chromosome transcript were translated, marked at the bottom of the alignment, would include the two conserved domains (HEAT and CND3) but lack the less conserved C-terminus region, the latter being absent in some species. (**b**) Alignment of the HEAT domain region. The arrowhead points amino acid 215 which is R in *E. plorans* 0B, likewise in the grasshoppers *L. migratoria* and *Ch. mollis*, but C in the *E. plorans* B chromosome sequence.

It was remarkable that in spite of the premature stop codon leading to transcript truncation, the predicted B-CAP-G protein for the B24 gene copies would include all conserved regions described for this protein, i.e. the HEAT domain, from exon 3 to exon 5, and the CND3 domain, from exon 11 to exon 17. An alignment of CAP-G protein sequences from several organisms showed that the C-terminus is the least conserved region and is even absent in most species, with the exception of the three grasshoppers species (*Ch. mollis*, *L. migratoria* and *E. plorans*) and *Drosophila melanogaster* (Fig. 4a). In all cases, the HEAT domain was highly conserved (Fig. 4b). Analysis with PROVEAN software for possible effects of the two putatively impairing substitutions (Arg-Cys substitution and stop codon) scored −3.1 for the substitution and −10.1 for the stop codon. These are under the −2.5 threshold, indicating that both changes are probably deleterious for the canonical *CAP-G* function, reinforcing the pseudogenic character of the *B-CAP-G* sequence.

### The B-CAP-G pseudogene is fragmented and transcribed in the B2 chromosome found in a different population

**Figure 5:**
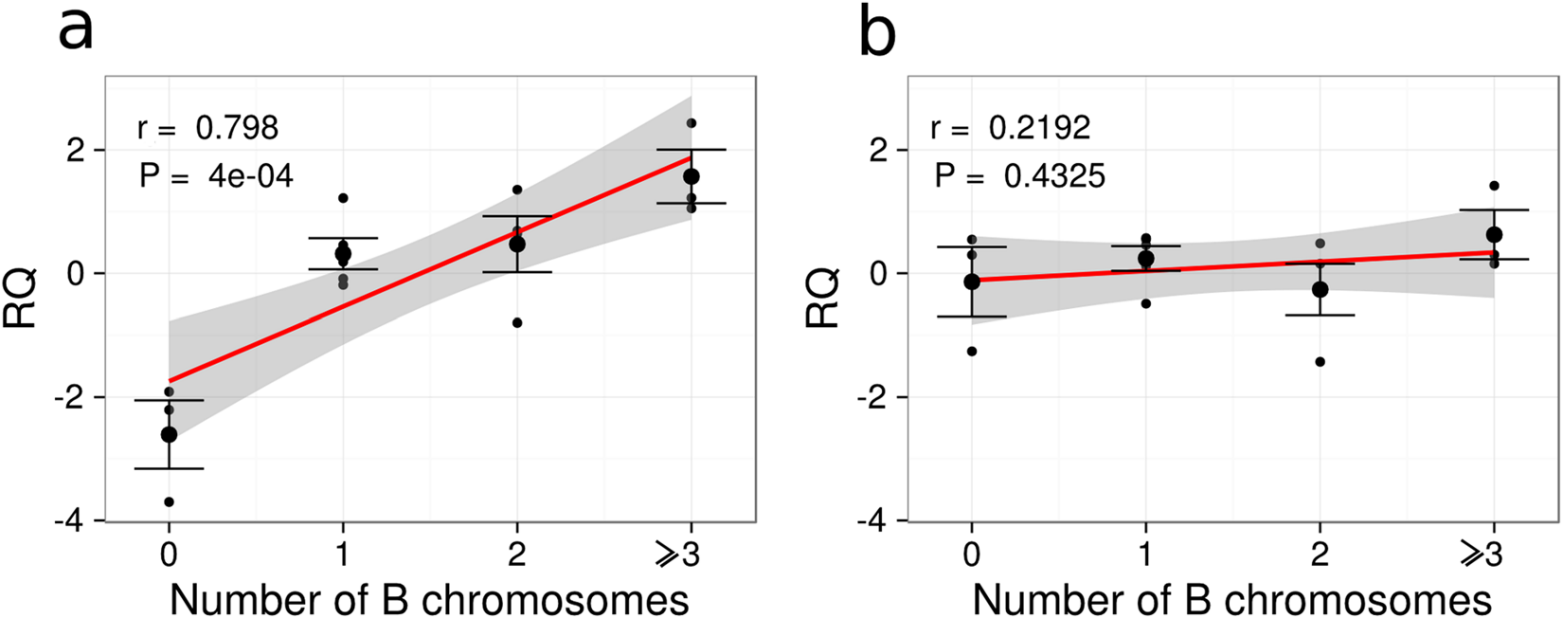
Relative quantification of *CAP-G* gene abundance by means of qPCR analysis on gDNA from males with different numbers of B2 chromosomes collected at the Salobreña population, using primers anchored on exon 18 (**a**) and exon 22 (**b**). Note that CAP-G abundance increases linearly with the number of B2 chromosomes when assayed for exon 18 (**a**), indicating that this exon is present in the B2 chromosome gene copies, but not for exon 22 (**b**), suggesting the absence of this exon in the B2 chromosome copies. Taken together, both experiments reveal the presence of truncated *CAP-G* gene copies in the B2 chromosome. RQ = relative quantity, r = Pearson’s linear correlation coefficient, P = P-value for Pearson’s correlation.

qPCR analysis of *CAP-G* abundance on gDNA from Salobreña males showed that it increased linearly with B2 chromosome number when we used primers anchored on exon 18 (Fig. 5a, Table S1), but not when primers were anchored on exon 22 (Fig. 5b, Table S1). This suggests the presence of truncated versions of the *CAP-G* gene (*B-CAP-G* pseudogene) in the B2 chromosome.

**Figure 6:**
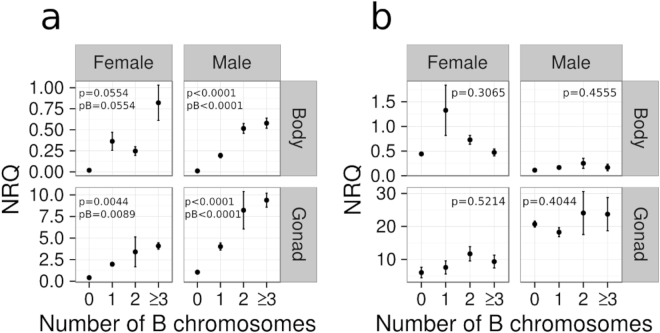
Relative quantification of *CAP-G* transcript abundance by means of qPCR analysis on cDNA from males and females with different numbers of B2 chromosomes collected at the Salobreña population, using primers anchored on exon 18 (**a**) and exon 22 (**b**). Note that CAP-G expression increased with B2 chromosome number when assayed for exon 18 (**a**) but not for exon 22 (**b**), suggesting the active transcription of B2 chromosome truncated gene copies. NRQ = normalized relative quantities; P = P-value for one-way ANOVA analysis; pB = Sequential Bonferroni P-value.

Similar qPCR experiments on cDNA from Salobreña individuals showed significant positive association between *CAP-G* transcript abundance and B chromosome number for exon 18 primers (Fig. 6a, Table S2) but not for exon 22 ones (Fig. 6b, Table S2). This was true both in comparisons of sex and of body parts. This pattern suggests that the excess of *CAP-G* transcripts observed in B-carrying individuals is due to active transcription of the *B-CAP-G* pseudogene in the B2 chromosome.

### Transcription of CAP-D2 and CAP-D3 is not altered by B2 or B24 chromosome presence or B-CAP-G transcription

**Figure 7:**
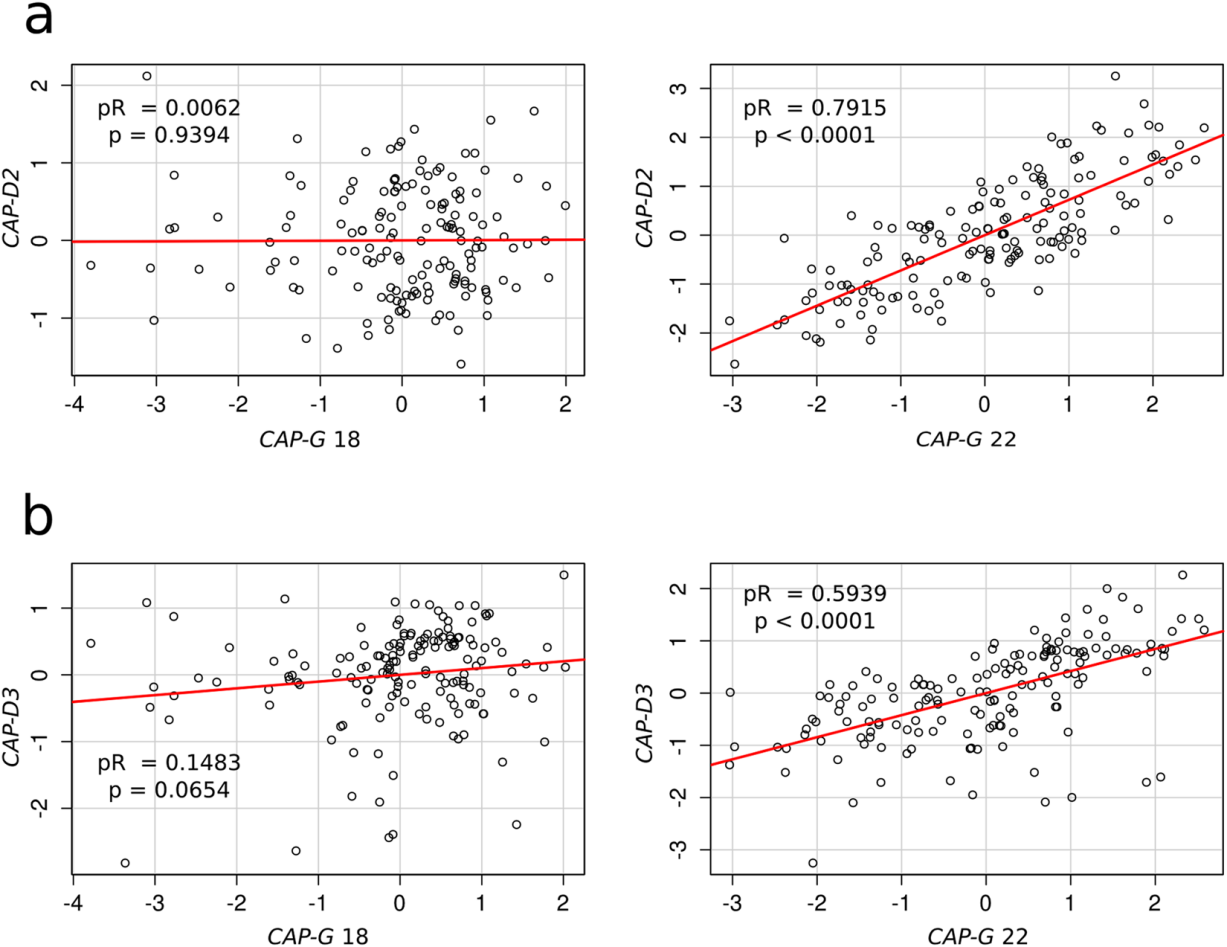
Partial regression plot showing the relationship of *CAP-G* transcription with the expression of *CAP-D2* (**a**) and *CAP-D3* (**b**) condensin I and II, respectively, subunit genes. Note that *CAP-G* transcription measured at exon 22 was positively correlated with *CAP-D2* and *CAP-D3* transcription levels, whereas that measured at exon 18 (which includes the *B-CAP-G* transcripts) was not. pR = partial correlation coefficient, p = p-value.

ANCOVA revealed that *CAP-D2* and *CAP-D3* transcription levels show significant differences between populations and sexes, but they are not influenced by the presence of B chromosomes (Tables 1 and 2). In addition, it showed that transcription levels of both *CAP-D2* and *CAP-D3* are significantly associated with *CAP-G* expression when measured at exon 22 (which is present only in A chromosomes) but not when measured at exon 18 (which is present in both A and B chromosomes). Likewise, multiple linear regression analysis showed that the relationship of *CAP-G* transcription with *CAP-D2* (condensin I) and *CAP-D3* (condensin II) transcription was only significant for *CAP-G* when measured at exon 22, i.e. for A chromosome *CAP-G* transcripts (Fig. 7; Tables S3 and S4). Taken together, these results indicate that the excess of *CAP-G*-like transcripts derived from the transcription of *B-CAP-G* pseudogenes does not alter the transcription rate of other condensin subunit genes.

**Table 1:** ANCOVA for *CAP-D2* transcription level (dependent variable).

Item	SS	df	MS	F	p
Intercept	36.69109	1	36.69109	198.5950	**0.000000**
CAP-G (ex. 18)	0.52497	1	0.52497	2.8415	0.094152
CAP-G (ex. 22)	15.64933	1	15.64933	84.7039	**5.55E-16**
pop	3.63684	1	3.63684	19.6848	**1.87E-05**
sex	15.10622	1	15.10622	81.7642	**1.33E-15**
bodypart	0.01699	1	0.01699	0.0920	0.762150
Bpre	0.34359	1	0.34359	1.8597	0.174911
pop*sex	1.48797	1	1.48797	8.0538	**0.005237**
pop*bodypart	0.64508	1	0.64508	3.4916	0.063833
sex*bodypart	0.18564	1	0.18564	1.0048	0.317938
pop*Bpre	0.01003	1	0.01003	0.0543	0.816112
sex*Bpre	0.00329	1	0.00329	0.0178	0.894086
bodypart*Bpre	0.10585	1	0.10585	0.5729	0.450418
pop*sex*bodypart	0.21997	1	0.21997	1.1906	0.277136
pop*sex*Bpre	0.19324	1	0.19324	1.0459	0.308261
pop*bodypart*Bpre	0.16818	1	0.16818	0.9103	0.341725
sex*bodypart*Bpre	0.15000	1	0.15000	0.8119	0.369149
pop*sex*bodypart*Bpre	0.03587	1	0.03587	0.1942	0.660164
Error	25.12645	136	0.18475		

*CAP-G* expression at exons 18 and 22 (continuous independent variables), and population (pop), sex, body part and B chromosome presence (Bpre) (discrete independent variables). SS = sum of squares, df = degrees of freedom, MS = mean sum of squares, p = p-value. Significant effects (p < 0.05) are noted in bold-type letter.

**Table 2:** ANCOVA for *CAP-D3* transcription level (dependent variable).

Item	SS	df	MS	F	p
Intercept	7E-05	1	7.2E−05	0.0002	0.99038
CAP-G (exon 18)	0.7539	1	0.75388	1.5368	0.2172
CAP-G (exon 22)	8.1258	1	8.12576	16.564	**7.9E−05**
pop	0.2207	1	0.22069	0.4499	0.50352
sex	5.0833	1	5.08332	10.362	**0.0016**
bodypart	0.0296	1	0.02954	0.0602	0.8065
Bpre	0.1961	1	0.19611	0.3998	0.52826
pop*sex	0.9846	1	0.98455	2.007	0.15883
pop*bodypart	1.0837	1	1.08372	2.2091	0.13948
sex*bodypart	0.016	1	0.01597	0.0326	0.85706
pop*Bpre	0.4588	1	0.45883	0.9353	0.33518
sex*Bpre	0.0074	1	0.00739	0.0151	0.90251
bodypart*Bpre	0.1848	1	0.18477	0.3767	0.54041
pop*sex*bodypart	0.0809	1	0.08094	0.165	0.68523
pop*sex*Bpre	0.1913	1	0.19129	0.3899	0.53337
pop*bodypart*Bpre	0.0033	1	0.00325	0.0066	0.93523
sex*bodypart*Bpre	0	1	0	0	1
pop*sex*bodypart*Bpre	0.2005	1	0.20048	0.4087	0.52371
Error	67.698	138	0.49057		

*CAP-G* expression at exons 18 and 22 (continuous independent variables), and population (pop), sex, body part and B chromosome presence (Bpre) (discrete independent variables). SS = sum of squares, df = degrees of freedom, MS = mean sum of squares, p = p-value. Significant effects are noted in bold-type letter.

## Discussion

Our present results, along with previous findings by Navarro-Domínguez *et al*.^24^, have shown that B chromosomes from two different populations (Torrox and Salobreña) carry several copies of the *B-CAP-G* pseudogene, as evidenced by physical mapping of the *CAP-G* gene on the B24 chromosome. In spite of growing evidence for the presence of protein-coding genes in B chromosomes, successful physical mapping of these genes on B chromosomes had only been achieved by means of BAC-FISH for the proto-oncogene c-kit gene in canids^17^, several genes in two species of cervids^39^ and chiclid fish^40^, and three genes (ScKIF4A,ScSHOC1 and ScAGO4B) in rye^23^ by conventional FISH.

Given the importance of the *CAP-G* gene for cell division, when the evolutionary fate of B chromosome is at stake, it is conceivable that the transcription of B chromosome *B-CAP-G* pseudogenes might change gene regulation equilibrium altering some cell division functions. These might facilitate B chromosome transmission advantage and maintenance in natural populations. Some effects hitherto described for B chromosome presence in *E. plorans* are reminiscent of the effects described for condensin mutants, and could thus be derived from the expression of the *B-CAP-G* pseudogene. For instance, B chromosome presence decreases egg fertility (i.e. the proportion of fertilized eggs)^41–43^ whereas, in *Drosophila*, some *CAP-G* mutants result in female sterile phenotypes^44^, and others provoke early mortality in embryos^9^. Moreover, it has been observed that B chromosome presence leads to an increase in chiasma frequency, and thus recombination, on A chromosomes^45^. This effect could actually be a byproduct of anomalous activity of the condensin complex in B carrying individuals; likewise, condensin mutants show higher rates of crossover in *C. elegans* ^8^.

Interestingly, Navarro-Domínguez *et al*.^24^ found a silenced truncated version of topoisomerase IIα (*B-TOP2A*) in the B24 chromosome of *Eyprepocnemis plorans*, and the *TOP2A* gene showed up-regulation in ovaries of B-carrying females (Navarro-Domínguez *et al*., submitted). The *TOP2A* gene shows a strong functional relationship with condensins^46^, and is involved in the resolution of ultrafine anaphase bridges^47^. The ins and outs of possible interactions between the *B-TOP2A* and *B-CAP-G* pseudogenes merits further research as it might shed some light on how B chromosomes managed to invade and persist in natural populations.

The possibility that the excess of *CAP-G* transcripts due to *B-CAP-G* pseudogene expression causes an enhancement in condensin function is actually remote because 1) the *B-CAP-G* transcripts carry a severe non-synonymous substitution and a premature stop codon, and 2) transcript levels of other condensin subunit genes (*CAP-D2* and *CAP-D3*) are independent on *B-CAP-G* expression level, according to our ANCOVA and multiple regression analysis. More indirectly, previous work has reported that an excess of *CAP-H2* leads to altered chromosome structure, dispersal of centromeres, chromosome unpairing and separation of salivary gland polytene chromosomal components^48,49^. No such symptoms have been observed in B carrying individuals of *E. plorans*. General hypercondensation of mitotic chromosomes and prophase shortening, as described for a gain of function mutation of *CAP-D3* ^50^, have neither been observed in *E. plorans*.

However, the lack of the last five exons does not necessarily mean that the predicted B-CAP-G protein cannot perform the CAP-G function since. In *Drosophila*, the N-terminal two-thirds of CAP-G are sufficient for assembling with the condensin I complex and efficient chromatin localization during mitosis, whereas the C-terminus is dispensable for condensin I function during cell cycle and development, although it is required for nuclear location and heterochromatinization during interphase^51^. Nevertheless, the amino acid change in position 215 (within the HEAT domain) of the predicted protein for the *B-CAP-G* pseudogene could have a high impact on the functionality of the predicted protein product, since most of the loss-of-function mutations hitherto described for this protein took place within the HEAT domain, which is also the most conserved region^52^. It is thus highly likely that the predicted protein for the *B-CAP-G* transcript is not fully functional. Even in this case, it is known that pseudogenic proteins could affect the activity of the parental proteins (for review, see Poliseno *et al*.^53^).

Even if the *B-CAP-G* transcripts were not translated, and considering that transcription rates of *CAP-D2* and *CAP-D3* are not altered by *B-CAP-G* transcription, the presence of anomalous *CAP-G* transcripts could influence the expression of the canonical *CAP-G* gene post-transcriptionally and, consequently, its normal functioning. There is growing evidence for a role of pseudogenes in the regulation of parental gene expression by means of several post-transcriptional levels of regulation, e.g. via epigenetic modification^53^, through the generation of endogenous siRNA^54^ or else acting as competitive inhibitors for binding to microRNAs^55^, the translational complex or other RNA-binding proteins^53^.

We cannot exclude the possibility that *B-CAP-G* expression is simply transcriptional noise, and therefore, it could lack further consequences other than the waste of energy employed to generate useless transcripts or polypeptides. Interestingly, the B24 variant in *E. plorans* harbors genes involved in cell division control and checkpoints. Some of them show a full-length CDS and are actively transcribed, suggesting a possible implication of B chromosome gene content in its own evolutionary success^24^. Among the possible implications of the presence of *B-CAP-G* transcripts mentioned above, the partial inhibition of normal *CAP-G* function through some kind of A chromosome mRNA neutralization, is highly consistent with the parasitic model of B chromosome evolution, as a decrease in *CAP-G* function might avoid the complete silencing of the B chromosome, thus allowing the expression of those B chromosome genes being important for its own survival. Of course, the mechanisms by which this A and B chromosome cross-talk takes place remain to be uncovered, but our present research opens new avenues for future research on such an interesting prospect.

## Methods

### Materials

This study was carried out on 80 adult individuals of the grasshopper *Eyprepocnemis plorans*, collected in Salobreña (Granada, Spain) and Torrox (Málaga, Spain) in October 2013, and showing different numbers of B chromosomes (Table 3). Embryos were obtained from egg pods laid by gravid females collected at Torrox, dissected in insect saline solution after ten days of incubation at 28 °C, which were used for *CAP-G* physical mapping.

**Table 3:** Individuals of *Eyprepocnemis plorans* analyzed per population, sex and number of B chromosomes.

Population	Sex	Bs	N
Salobreña	Female	0	2
B2 type		1	5
		2	2
		≥3	5
		Total	14
	Male	0	4
		1	10
		2	4
		≥3	3
		Total	21
Torrox	Female	0	4
B24 type		1	9
		2	8
		≥3	1
		Total	22
	Male	0	2
		1	7
		2	8
		≥3	6
		Total	23
Total Samples			80

Bs = Number of B chromosomes, N = Number of individuals.

Testes and ovaries were dissected from anesthetized animals. One gonad (testis or ovary) and the somatic body were immediately frozen in liquid nitrogen and stored at −80 °C until DNA and RNA extraction. The other testis was fixed in 3:1 ethanol-acetic acid and stored at 4 °C for cytological analysis. The remaining ovary was immersed in 2% colchicine in isotonic insect saline solution for 2 hours, fixed in 3:1 ethanol-acetic acid, and stored at 4 °C for cytological analysis. In males, the number of B chromosomes was determined by visualizing them in primary spermatocytes at diplotene or metaphase I obtained by squashing two testis tubules in a drop of 2% lactopropionic orcein^56^. In females, the number of B-chromosomes was analyzed in squash preparations of two ovarioles submitted to C-banding, a technique which shows B chromosomes much darker than the A chromosomes^56^. For physical mapping of the *CAP-G* gene, we used 0.05% colchine-treated embryos prepared as described in Camacho *et al*.^56^. In embryos, B chromosome presence was determined by staining chromosome slides with 2 μg/ml 4′,6 diamidino-2-phenylindole (DAPI) which reveals the presence of several large DAPI ^+^ bands on the B chromosome.

### Nucleic acids extraction and cDNA synthesis

Genomic DNA (gDNA) from Salobreña males was extracted using the GenElute Mammalian Genomic DNA Miniprep kit (Sigma). Absence of degradation was checked in a 1% TBE-agarose gel, and quantification and assessment of 260/280 and 260/230 ratios were performed with an Infinite M200 Pro NanoQuant (Tecan). For qPCR working solution, gDNA samples were diluted to 5 ng/μl.

Total RNA extractions were performed using the Real Total RNA Spin Plus kit (Durviz) for somatic bodies and the RNeasy Lipid Tissue Mini Kit (Qiagen) for gonads, complementing both protocols with a DNase treatment on the column membrane (20 units of Amplification Grade DNase I (Sigma) for 30 minutes). gDNA contamination on gonad extracted RNA was negligible or nonexistent, but the RNA extracted from bodies needed an additional DNase treatment with the REALSTAR kit (Durviz). Quality check and quantification of total RNA was performed with a Tecan’s Infinite 200 NanoQuant and in a denaturing MOPS-agarose gel to assure the absence of RNA degradation and DNA contamination, which was further corroborated by lack of PCR amplification of ribosomal DNA (rDNA) and histone genes on the extracted RNA. For this purpose, we used the 18SE and 1100 R primers^57^ with PCR conditions described in Ruiz-Estévez *et al*.^29^, and the H3 gene primers described in Colgan *et al*.^58^. Retrotranscription was performed on 100 ng total RNA combined with random and oligo-dT hexamers from the PrimeScriptTM RT reagent - Perfect Real Time-Kit (Takara), and 1:10 diluted to get the working solution.

### Chromosomal location of *CAP-G* gene using Tyramide-coupled FISH

Physical mapping of the *CAP-G* gene was performed on embryo chromosomes, following the protocols for fluorescent *in situ* hybridization coupled with tyramide signal amplification (FISH-TSA) described previously by Krylov *et al*.^59,60^ and Funkhouser-Jones *et al*.^61^ with minor changes (Supplementary Materials and Methods).

To prepare the probes for FISH, two fragments of the CAP-G transcript were amplified in *E. plorans* cDNA, using the following primer pairs designed with Primer3^62,63^: F: 5′-AAGATGAGAAGTCACCCGTTGT-3′ and R: 5′-TCTAATGCCTGGATCTCTGGTT-3′, which amplifies a 1355 bp fragment comprising from exons 2 to 10; and F: 5′-AATGATCCATTCACACTCACCA-3′ and R: 5′-CAGGAGAAGCTTTGCTTTGATT-3′, which amplifies a 1420 bp fragment comprising from exons 11 to 19. PCR reaction was performed with the Horse-PowerTaq DNA polymerase (Canvax) kit, and contained 1X PCR buffer, 2.5 mM MgCl_2_, 0.25 mM dNTPs, 0.4 μM of each primer, 2.5 units of DNA polymerase and about 5 μl of cDNA (obtained as described below) per reaction. The thermocycler program was as follows: an initial denaturation step at 94 °C for 5 min, 40 cycles at 94 °C (30 s), 60 °C (30 s) and 72 °C (2 min), and a final elongation step at 72 °C for 8 min. These two fragments were amplified separately and PCR products were labelled for their use in FISH-TSA (Supplementary Material and Methods). The FISH experiment was performed with the two probes simultaneously.

### Prediction of B-CAP-G sequence functionality

To analyze sequence variation in *B-CAP-G* pseudogene in respect to the *CAP-G* gene located in the A genome, we used SSAHA2^64^ to map Illumina reads obtained from 0B and 1B *E. plorans* female RNA, and those obtained from male genomic DNA with 0B and 4B from the Torrox population (NCBI-SRA accession numbers SRR2969416, SRR2969417, SRR2970625 and SRR2970627^24^), using as reference the sequence of the *E. plorans CAP-G* transcript described in Navarro-Domínguez *et al*.^24^ and available on GenBank under accession number KX034166. To search for B-specific sequence variants, we quantified the number of reads carrying a given nucleotide at each position using pysamstats (https://github.com/alimanfoo/pysamstats), and selected the positions where the variants were shared by the two + B libraries but were absent in the two 0B libraries. For further confirmation, we also mapped reads obtained from three different 0B females, three 0B males and three 4B males, also from the Torrox population (Martín-Peciña *et al*., personal communication).

Although *B-CAP-G* lacks five exons on the 3′ end (Fig. 2)^24^, it has been reported that condensin can be functional even with a C-terminal deletion^51^, for which reason we compared the predicted amino acid sequence for the *B-CAP-G* transcript with homologous proteins available in the databases. We got 18 sequences of CAP-G condensin I subunit in the NCBI-GenBank database from many different organisms (seven mammals, a bird, an amphibian, a fish and eight insects; see Table S5 for accession numbers). To compare with other grasshopper species, we used the *CAP-G* transcript from a *de novo* assembled transcriptome of *Locusta migratoria* (Ruiz-Ruano *et al*., in preparation) and from a Trinity-based *de novo* assembly performed by us using Illumina reads of *Chortippus mollis* found in NCBI-SRA database (Accession number SRR2051368^65^), and predicted the protein coded by those transcripts (Table S5). Alignments were performed using Geneious 4.8^66^. The possible impact of amino acid changes and C-terminus deletion was predicted with PROVEAN^67^.

### Search for *E. plorans CAP-D2* and *CAP-D3* transcript sequences

In order to analyze whether the expression of the *B-CAP-G* pseudogene influence transcription levels of other subunits of the condensin complex, we obtained the sequences of *CAP-D2* and *CAP-D3* transcripts and designed primers for qPCR experiments. Those sequences were obtained by local TBLASTN^68^ of *CAP-D2* and *CAP-D3* protein sequences described for *Zootermopsis nevadensis* from NCBI-GenBank (accession numbers KDR15738.1 and KDR16504.1) on the *de novo* assembled transcriptome of *E. plorans* ^69^. Sequences of *E. plorans CAP-D2* and *CAP-D3* transcripts can be found in NCBI-GenBank under accession numbers KX376471 and KX376472, respectively.

### Quantitative PCR (qPCR)

*Experiment and primer design*. In order to test whether *B-CAP-G* is located and fragmented in the B2 chromosome from the Salobreña population, we performed relative quantification of the abundance of the *CAP-G* gene by means of qPCR analysis, using two primer pairs, one anchored on exon 18, which is expected to amplify on gDNA from both A and B chromosomes, and the other anchored on exon 22, which is expected to amplify only on A chromosome (Fig. 2). This is based on previous observations in the Torrox population indicating that *B-CAP-G* lacks the last five 3′ exons (20 to 24) (Fig. 2)^24^. We also PCR amplified these regions on cDNA from gonads and somatic bodies of males and females with different numbers of B2 chromosomes from the Salobreña population, in order to ascertain whether *B-CAP-G* is transcribed in this population. Primers for *E. plorans CAP-G* gene were F: 5′ -GAGGTATGGAACACGCACAA-3′ and R: 5′-AGTGGCACGTTTCGTCTTCT-3′, anchored in exon 18, and F: 5′-CAACAGCGCCTGTCACTAAA-3′ and R: 5′-GCTGAGGTGTCTGCTCACAA-3′, anchored in exon 22 (Fig. 2), as reported in Navarro-Domínguez *et al*.^24^.

In addition, we analyzed both populations (Torrox and Salobreña) for whether the expression of the *B-CAP-G* pseudogene in B-carrying individuals influences the transcription of genes for other subunits of the condensin complex, specifically *CAP-D2* (Condensin I complex subunit D2) and *CAP-D3* (Condensin II complex subunit D3). Primer sequences for *E. plorans CAP-D2* and *CAP-D3* were designed with Primer3 software^62,63^. They were F: 5′-GGTGACATTGCTTTCCGATT-3′ and R: 5′-CTCCGGATGTGCATCTGTTA-3′ for *CAP-D2*, and F: 5′-CCCAGAAAGAAGCTGAGGTG-3′ and R: 5′-TCAAAACATGCCTACCAGCA-3′ for *CAP-D3*.

*Selection of reference genes*. According to the results of validation and stability analysis of reference genes in *E. plorans* described in Navarro-Domínguez *et al*.^70^, two or three reference genes were employed here, specifically *Act-RP49* for somatic tissue and ovaries from Salobreña females, *GAPDH-Tub* for somatic tissue and *Act-Tub-Arm* for testes from Salobreña males, *RP49-Act-Arm* for somatic tissue and *Act-Arm* for ovaries from Torrox females, and *Act-Tub* for somatic tissue and *Act-RP49* for testes from Torrox males.

*qPCR reaction*. qPCR amplification was performed in a Chromo4 Real Time PCR thermocycler (BioRad). Reaction mixture contained 5 μl gDNA or cDNA, 5 μl SensiMix SYBR Kit and 2.5 μl each 2.5 μM primer in a total 15 μl volume. Electronic pipettes (Eppendorf Research ® Pro) were used in order to minimize pipetting errors, and each reaction was carried out in duplicate. Samples were discarded when the coefficient of variation between technical replicates was higher than 5%. Quantitative PCR protocol consisted in an initial denaturalization step for 10 min at 95 °C, followed by 40 cycles of 15 s at 94 °C, 15 s at annealing temperature (60 °C for *CAP-G* and 58 °C for *CAP-D2* and *CAP-D3*) and 15 s at 72 °C, with plate reading at the end of every cycle. Specificity of reaction was assessed for each primer pair by means of a dissociation curve (from 72 °C to 95 °C with plate reading every 1 °C) after the 40th cycle. Fluorescence was measured and processed using Opticon Monitor 3.1 (Bio-Rad Laboratories, Inc). Negative controls for each primer pair were included in all reactions.

*qPCR data analysis*. We calculated efficiency for all primer pairs, by a standard curve performed with 1:10 serial dilutions. Relative Quantities (RQs) were calculated referred to a calibrator sample, following the same method described in Navarro-Domínguez *et al*.^70^. In cDNA reactions, RQ values were normalized by the geometrical average of the most stable reference genes for each sample type^71^.

In order to meet the normality requirements of parametric analyses, we transformed the qPCR data to natural logarithms. We tested the linear relationship between B chromosome number and abundance of the *CAP-G* gene (with primer pairs anchored on exons 18 and 22), in *E. plorans* males from Salobreña, using Pearson’s correlation analysis. *CAP-G* expression level in bodies and gonads of males and females with different numbers of B chromosomes was tested by one-way ANOVA and post-hoc sequential Bonferroni correction. To test whether *CAP-G* transcription is associated with *CAP-D2* and *CAP-D3* transcription, we performed an analysis of covariance (ANCOVA) including population, sex, body part and B chromosome presence as discrete independent variables, CAP-G expression levels measured in exon 18 and in exon 22, as continuous independent variables, and CAP-D2 or CAP-D3 expression levels as dependent variables. We finally calculated partial correlations of the expression levels of CAP-G (exon 18) and CAP-G (exon 22) with the expression levels of CAP-D2 or CAP-D3 by means of multiple regression analysis.

## Electronic supplementary material

Supplementary Tables and Methods
