# Supplementary material for: Transcription of a B chromosome CAP-G pseudogene does not influence normal Condensin Complex genes in a grasshopper
Source: Sci Rep. 2017 Dec 15;7:17650. doi: 10.1038/s41598-017-15894-5 (PMC5732253; doi:10.1038/s41598-017-15894-5)
Supplement: Supplementary file 1 — Supplementary Tables and Methods [file 41598_2017_15894_MOESM1_ESM.pdf]

SUPPLEMENTARY TABLES AND METHODS

Transcription of a B chromosome *CAP-G* pseudogene does not influence normal Condensin Complex genes in a grasshopper

Beatriz Navarro-Domínguez[1,2], Francisco J. Ruiz-Ruano[1], Juan Pedro M. Camacho[1], Josefa Cabrero[1], María Dolores López-León[1,]*

[1]Departamento de Genética. Facultad de Ciencias, Universidad de Granada.18071 Granada, Spain

[2]Department of Ecology, Evolution, and Organismal Biology, Iowa State University, Ames, Iowa, USA

* Correspondence and requests for material should be addressed to M.D.L.L.

(email:mdlopez@ugr.es)

Supplementary Tables

Item	r	t	df	p
CAP-G (ex. 18)	0.7980	47.741	13	**0.0004**
CAP-G (ex. 22)	0.2192	0.81011	13	0.4325

Table S1. Pearson's linear correlation analysis between the number of B2 chromosomes and *CAP-G* abundance analyzed by qPCR in gDNA of *E. plorans* males (N=15) from Salobreña. Note that *CAP-G* abundance was measured separately for exon 18 (which is present in both A and B chromosomes) and exon 22 (only present in A chromosomes). r= linear correlation coefficient, t= Student test, df= degrees of freedom, p= p-value. Significant effects are noted in bold-type letter.

Exon	Sex	Body part	df	SS	MS	F	p	pB
18	Female	Body	3	1.1788	0.3929	3.555	0.0554	**0.055**
		Gonad	3	23.726	7.9088	8.364	0.0044	**0.009**
	Male	Body	3	0.8373	0.2791	29.81	4E−07	**2E−06**
		Gonad	3	159.94	53.313	18.02	1E−05	**3E−05**
22	Female	Body	3	2.1851	0.7284	1.374	0.3065	
		Gonad	3	40.459	13.486	0.801	0.5214	
	Male	Body	3	0.0457	0.0153	0.911	0.4555	
		Gonad	3	114.72	38.239	1.038	0.4044	

Table S2. One-way ANOVA analyses comparing *CAP-G* transcription level between *E. plorans* individuals from Salobreña with different number of B2 chromosomes. Note that transcription level was separately measured at exon 18 (being present in both A and B chromosomes) and exon 22 (only present in A chromosomes). df= degrees of freedom, SS= sum of squares, MS= mean sum of squares, p= p-value, pB=sequential Bonferroni p-value. Significant effects are noted in bold-type letter.

Exon	β	Partial	R-square	$t_{(151)}$	p
CAP-G (ex. 18)	0.0043	0.0062	0.56588	0.0761	0.9394
CAP-G (ex. 22)	0.8887	0.7915	0.56588	15.914	**4E−34**

Table S3. Partial and semipartial correlation between *CAP-G* transcription levels, measured at exons 18 and 22, on the expression of the *CAP-D2* subunit gene of condensin. β= regression coefficient, t= Student t test (degrees of freedom in brackets), p= p-value. Significant effects are noted in bold-type letter.

**Exon**	**β**	**Partial**	**R-square**	**$t_{(153)}$**	**p**
CAP-G (ex. 18)	0.138	0.1484	0.55087	1.8556	0.06544
CAP-G (ex. 22)	0.6793	0.5939	0.55087	9.1313	**4E−16**

Table S4. Partial and semipartial correlation between *CAP-G* transcription levels, measured at exons 18 and 22, on the expression of the *CAP-D3* subunit gene of condensin II. β= regression coefficient, t= Student t test (degrees of freedom in brackets), p= p-value. Significant effects are noted in bold-type letter.

Organism	Accession Number
Homo sapiens	NP_071741
Pan troglodytes	XP_526535
Macaca mulatta	XP_001102882
Bos taurus	NP_001095846
Canis lupus familiaris	XP_536278
Rattus norvergicus	XP_223468
Mus musculus	NP_062311
Gallus gallus	XP_420769
Xenopus tropicalis	NP_989190
Danio rerio	NP_001243134
Drosophila melanogaster	NP_001163135.1
Acromyrmex echinatior	XP_011050712
Solenopsis invicta	XP_011166263
Megachile rotundata	XP_003705431
Bombus impatients	XP_003488800
Apis florea	XP_003696218
Chorthippus mollis	KX905139
Locusta migratoria	KX905140

Table S5. NCBI-GenBank accession numbers of CAP-G protein sequences used for prediction of the *B-CAP-G* sequence functionality

Supplementary Methods

Chromosomal location of *CAP-G* gene using Tyramide-coupled FISH (Supplementary information)

200 ng each of two CAP-G fragments probes were labeled with digoxigenin-11-dUTP (Roche) by random primers using Decalabel DNA labeling kit (Thermo Scientific) according to manufacturer's recommendation but using 9 µl of a labeled dNTP mix containing 1mM dATP, 1 mM dCTP, 1 mM dGTP, 0.65 mM dTTP and 0.01 mM digoxigenin-11-dUTP. Probe was labeled at 37 ºC for 20 hours and then purified and resuspended in 10 µl ultrapure water.

Chromosome preparations were dehydrated in 70 %, 90 % and 100 % ethanol series for 3, 3 and 5 min, respectively. Afterwards, they were air dried and stored at 60 ºC overnight. After incubation with 200 µl RNase (100 µg/ml in 2XSSC, 300 mM NaCl, 30 mM sodium citrate, pH 7.0) for 2 hours at 37 ºC, they were washed three times in 2XSSC for 5 min and once in PBS (phosphate buffer saline, pH=7.3) for 5 min. Metaphase spreads were then treated with 4% paraformaldehyde for 10 min at room temperature followed by three washes in PBS for 5 min each. Endogenous peroxidases were quenched by treating slides for 30 min with 1 % H_2O_2, followed by three washes in PBS for 5 min and one in distilled water. Chromosome preparations were then dehydrated in a series of 70 %, 90 % and 100 % ethanol for 3, 3 and 5 min, respectively. Chromosomal DNA was denatured and hybridized with 50 µl of hybridization mixture, composed of 50 % deionized formamide, 2XSSC and 40 ng of each CAP-G labeled probe, under a plastic coverslip in a hot plate at 80 ºC for 6 min. Slides were then incubated in a humid chamber overnight at 37 ºC. Posthybridization washing and signal

detection of FISH-TSA were performed as reported by Krylov *et al.*[59,60]. For detection of digoxigenin-labeled CAP-G gene probe, we used an anti-digoxigenin antibody conjugated with horse-radish peroxidase (Sigma) at a concentration of 1:500 in TNB (Tris-NaCl-blocking buffer), by incubating slides for 1 hour in a humid chamber at room temperature. Tyramide signal amplification reaction was performed using TSA Plus Fluorescence kit (Perkin Elmer), applying 100 µl of 1:50 diluted tyramide solution per slide with dark-incubation for 10 min at room temperature. Slides were counterstained with 100 µl of 2 µg/ml DAPI (4`,6-diamidino-2-phenylindole) in McIlvaine's buffer (pH=7) for 18 min, washed for 5 min in PBS, 3 min in distilled water and mounted in antifading solution (Vectashield H-100). Hybridized metaphase cells were analyzed under a BX41 Olympus epifluorescence microscope and photographs were taken with a DP70 cooled camera. Images were merged and optimized for brightness and contrast with the Gimp software.
